# A Novel Reference Plasmid for the Qualitative Detection of Genetically Modified Rice in Food and Feed

**DOI:** 10.1155/2015/948297

**Published:** 2015-09-30

**Authors:** Liang Li, Mei Dong, Na An, Lixia Liang, Yusong Wan, Wujun Jin

**Affiliations:** Biotechnology Research Institute, Chinese Academy of Agricultural Sciences-Inspection and Testing Center for Environmental Risk Assessment of Genetically Modified Plant-Related Microorganism (Beijing), Ministry of Agriculture, No. 12 Zhongguancun South Street, Haidian District, Beijing 100081, China

## Abstract

Rice is one of the most important food crops in the world. Genetically modified (GM) technology has been used in rice to confer herbicide tolerance and pathogen or insect resistance. China invests heavily in research on GM rice. By the end of 2014, at least 250 transgenic rice lines had been developed in China. To monitor the presence of GM rice in food and feed, we collected information on foreign elements from 250 transgenic rice lines and found 5 elements, including the *Agrobacterium tumefaciens* nopaline synthase terminator (T-NOS), the cauliflower mosaic virus 35S promoter (CaMV35S), the ubiquitin gene (Ubi), the bar gene, and the hygromycin phosphotransferase gene (Hpt), that are commonly present in GM rice. Therefore, we constructed a novel plasmid (pBJGMM001) that contains fragments of these elements and two endogenous reference genes (the sucrose phosphate synthase gene, SPS, and the phosphoenolpyruvate carboxylase gene, PEPC). pBJGMM001 can serve as a standard for detecting 96% of GM rice lines in China. The primers, amplicons, reaction mixture, and PCR program were developed based on Chinese National Standards. The protocol was validated and determined to be suitable for practical use in monitoring and identifying GM rice.

## 1. Introduction

Rice (*Oryza sativa* L.), which is one of the most important cereal crops in the world, is a fundamental food source that provides nutrition for nearly half of the global population [1]. Genetic transformation of rice has progressed rapidly in recent decades to meet specific requirements, and a number of agronomically important traits, including enhancement of resistance to insects and diseases [2], tolerance to herbicides, quality improvement [3], and increased nutritional value [4], have been introduced into rice. Since the first transgenic rice plant was obtained by Toriyama et al. in 1988, transformation of rice has been an important issue in transgenic research in the field of modern agricultural biotechnology [5–7]. In August 2009, two insect-resistant rice varieties, Huahui 1 (TT51-1) and Bt Shanyou 63, obtained security certificates allowing their production in Hubei province. Therefore, China is likely to begin commercially cultivating GM rice very soon [8].

Rice is a staple crop, and many consumer concerns have arisen regarding the existence of transgenes in such crops and the food chain in general. Consumers are also concerned about the probable commercial release of GM crops in the future. GM rice varieties developed in China are soon to be approved for agricultural cultivation and production [9]. In China, rice accounts for more than 20% of the total planted area and high harvest yields of conventional rice are achieved through heavy use of herbicides and pesticides. The use of GM rice varieties conferring resistance to pests or tolerance to herbicides will help to reduce the use of chemicals for crop protection [10]. Although GM rice may increase the efficiency of modern agriculture and provide other benefits, not all global markets fully accept GM products for a variety of reasons, including the introduction of new allergens, the possible development of antibiotic-resistant bacterial strains, and the modification of environmental biodiversity [11]. To monitor and verify the presence and distribution of GM rice, there is a need for GM detection methods that are accurate, fast, and inexpensive. Various methodologies have been developed to analyze and/or detect the presence of GMOs in food products using PCR [12], enzyme-linked immunosorbent assays (ELISAs) [13], microarrays [14], and electrophoresis [15]. These DNA- or protein-based methods are versatile, sensitive, specific, and precise.

PCR has become the technique of choice for the determination of GMOs in a sample [16, 17]. PCR-based techniques that target well-chosen, specific transgenic DNA segments have been demonstrated to be among the most adequate and efficient methods for the qualification and quantification of transgenic crops. This is primarily due to the stable nature of the DNA molecule as well as the extreme sensitivity of PCR technology [18]. PCR methods depend on certified reference materials, which are used for the calibration or quality control of GMO measurements. Plasmids have been demonstrated to be a good alternative to reference materials for GMO detection [19, 20]. Currently, single- or multiple-target plasmids, containing either one GM-specific or endogenous sequence or several specific GM elements from one species, have been developed as reference materials [20–25]. However, few standard reference molecules containing GM elements from rice have been reported.

The aims of this study were to develop a reference plasmid for greater coverage of the possible GM ingredients in food or feed products using the following steps: (I) investigation of the status of transgenic rice in China, (II) construction and validation of a reference plasmid, and (III) application to practical sample analysis. Additionally, the novel standard reference molecule developed in this study, which includes rice endogenous reference genes and sequences of GM elements from rice, was demonstrated to be a valid substitute for certified positive reference materials in GM rice detection.

## 2. Material and Methods

### 2.1. Plant Samples and Reagents

To prepare samples containing the exogenous and endogenous target sequences, genuine seeds from the GM rice varieties TT51-1, Kangyou97, Kefeng6, Kefeng8, KMD1, Bar68-1, and Bar66-1 as well as nontransgenic rice seeds were collected by members of our laboratory. Plant genomic DNA was isolated from rice flour using the Wizard Magnetic DNA Purification System for Food according to the manufacturer's instructions (Promega, Madison, WI, USA). The DNA pellets were dissolved in 100 *μ*L of nuclease-free water. DNA quantification was performed using a PicoGreen assay (Quant-iT PicoGreen dsDNA Kit, Invitrogen, Carlsbad, CA, USA), and the samples were diluted to a 50 ng/*μ*L working stock, which was stored in aliquots at −80°C. Restriction enzymes were purchased from New England Biolabs (Ipswich, MA). DNA synthesis, primer synthesis, and DNA sequencing were performed by Genewiz, Inc. (Suzhou, China).

### 2.2. Construction of the pBJGMM001 Plasmid

To efficiently construct the plasmid, the exogenous and endogenous fragments were cloned into the* Eco*RV site of a pUC57 vector. We used validated PCR-based methods to amplify exogenous and endogenous genes using Chinese National Standard PCR primers (Table 1), amplification reaction mixtures (a 25 *μ*L reaction volume containing GoTaq Green Master Mix (2x) 12.5 *μ*L, each primer 0.5 *μ*L (10 *μ*M), DNA template (25 ng/*μ*L) 2.0 *μ*L, and ddH_2_O 9.5 *μ*L), and amplification cycling parameters (initial denaturation at 94°C for 5 min, 40 cycles of denaturation at 94°C for 30 sec, annealing at 58°C for 30 sec, extension at 72°C for 30 sec, and final extension at 72°C for 10 min and cooling to 4°C). The ampicillin-resistant plasmid was transformed into* Escherichia coli*, and the transformed strain was stored at −80°C. Five liters of Luria-Bertani (LB) medium containing 100 *μ*g/mL ampicillin was inoculated with 5 mL of a preculture of* E. coli* containing the plasmid pBJGMM001 and was shaken vigorously overnight at 37°C. The culture was then centrifuged at 4°C, and the plasmid DNA was isolated and purified using the PureYield Plasmid Midiprep System (Promega, Madison, WI, USA) according to the manufacturer's protocol. Plasmid samples were stored in Axygen tubes (1 ng/*μ*L pBJGMM001 in 500 *μ*L of buffer (1 mM Tris, 0.01 mM EDTA, pH 8.0); 10^6^ copies/*μ*L).

### 2.3. Purification and Characterization of the Reference Plasmid pBJGMM001

As a reference molecule, pBJGMM001 was processed as follows.

- Plasmid DNA isolated from the transformed* E. coli* cells was sequenced completely to verify that all target DNAs were present and correctly cloned.
- The concentration and purity of the plasmid DNA preparation were measured via UV spectrophotometry. The purity of the nucleic acid was assessed spectrophotometrically by comparing the UV absorbance of the sample at 260 nm to that at 280 nm. Taking into account the generally accepted mean extinction coefficients for double-stranded DNA at 260 nm and 280 nm, pure nucleic acid samples are expected to have an* A*260/*A*280 ratio of approximately 1.8 and an* A*260/*A*230 ratio above 2.0 [31].
- The purity of plasmid pBJGMM001 was analyzed by agarose gel electrophoresis.

## 3. Results and Discussion

### 3.1. Investigation of GM Rice Development in China

GM rice in China was investigated using the following tools: the Chinese National Knowledge Infrastructure database (http://www.cnki.net/), the Wanfang database (http://www.wanfangdata.com.cn/), scientific papers (Elsevier, Springer, ACS, and others), the GMO Detection Method database (http://gmdd.shgmo.org/), and Detection and Monitoring of GM Crops of China (http://www.gmcrop.cn/). We summarized the available information on exogenous genes, regulatory elements, and screening markers in GM rice. Based on this information, a statistical analysis was performed to select the genes that appear most frequently in GM rice.

When designing the novel plasmid, we aimed to use fewer elements to achieve greater detection coverage. As a first step toward this goal, we investigated the status of GM rice development in China from 1989 to 2014. Based on a database search, we determined that the total number of papers published on GM rice varieties is approximately 250 (Supplementary Table S1 in Supplementary Materials available online at http://dx.doi.org/10.1155/2015/948297). These varieties include TT51-1, which received a biosafety certificate, and other GM lines undergoing field trials and lines that are currently in the development stage in China. The investigation revealed a large number of commonly occurring transgenic elements, including the following: the cauliflower mosaic virus 35S promoter (CaMV35S), the* Agrobacterium tumefaciens* nopaline synthase terminator (T-NOS), the hygromycin phosphotransferase gene (Hpt), the ubiquitin gene (Ubi), the *β*-glucuronidase gene (GUS), the bar gene (Bar), the actin promoter (P-Act), the* Agrobacterium tumefaciens* nopaline synthase promoter (P-NOS), the neomycin phosphotransferase II gene (NPTII), and the cauliflower mosaic virus 35S terminator (T-35S).

Due to this large number of common elements, most of the GMO detection laboratories around the world have started performing initial PCR-based screens followed by more specific identification and quantification assays (when appropriate and required by legislation). The use of initial screens that target elements common to multiple GM events can facilitate rapid and cost-effective discrimination of GMO and GMO-free samples [32]. Based on the investigation results, we selected five exogenous genes (CaMV35S, T-NOS, Ubi, Bar, and Hpt) as the detection targets, which achieved detection coverage of 96% (Table 2).

### 3.2. Construction and Purification of pBJGMM001

**Figure 1:**
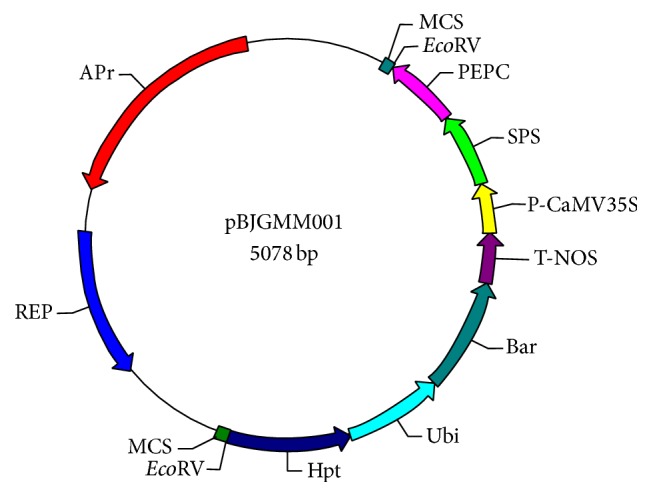
Schematic diagram of the integrated fragments in pBJGMM001. The image of the plasmid structure was generated using WinPlas 2.7 software (Rich Goldstein).

The pBJGMM001 plasmid contained DNA sequences from part of the rice taxon-specific SPS gene, the PEPC gene, and the exogenous elements CaMV35S, T-NOS, Ubi, Bar, and Hpt (Figure 1). The exogenous and endogenous gene fragments were cloned into the* Eco*RV site of the pUC57 vector. In vitro DNA synthesis of the entire target fragment saves time and is easier compared to traditional overlapping PCR. Using gene synthesis, researchers save money on the reagents needed for plasmid construction, cloning, and sequencing and can also save time by outsourcing the synthesis of gene sequences that are difficult to clone, thereby avoiding repeated cloning failures. Moreover, ~10 bp was added to both amplicons to increase the amplification efficiency.

Plasmid pBJGMM001 was fully sequenced by three independent laboratories. The results were consistent with expectations (data not shown). The sequence analysis did not reveal the presence of a mixed population of plasmids. The* A*260/*A*280 and* A*260/*A*230 ratios measured for the plasmid solution were 1.90 ± 0.04 and 2.12 ± 0.03, respectively, indicating sufficient DNA purity; however, such values do not exclude the possibility that traces of contaminating protein may be present. Because no smear was visible in the plasmid preparation and no RNA band was visible, it can be concluded that the plasmid preparation was not contaminated with external genomic DNA or a large amount of RNA (data not shown). However, traces of genomic DNA or RNA from host bacterial cells cannot be excluded in the final plasmid preparation. Such traces do not influence the detection of target sequences.

**Figure 2:**
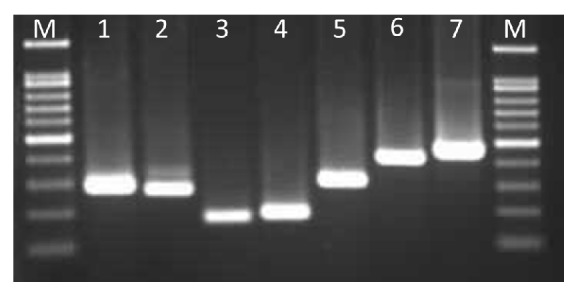
Validation of the specific sequences of endogenous rice reference genes and exogenous genes. The image shows 2.0% agarose gel electrophoresis of the amplification products obtained via PCR. M, ladder; lanes 1–7: SPS (287 bp; lane 1), PEPC (271 bp; lane 2), T-NOS (180 bp; lane 3), CaMV35S (195 bp; lane 4), Ubi (314 bp; lane 5), Bar (430 bp; lane 6), and Hpt (472 bp; lane 7).

Amplified fragments of SPS (287 bp), PEPC (271 bp), T-NOS (180 bp), CaMV35S (195 bp), Ubi (314 bp), Bar (430 bp), and Hpt (472 bp) were observed when pBJGMM001 DNA was used as a template for amplification (Figure 2). The primer pairs were designed to specifically amplify a region containing both a trait gene and a regulatory gene, such as a terminator.

### 3.3. Application Assay

In this study, to investigate the utility of pBJGMM001 in practical samples, we tested samples from all potential commercial GM rice lines, including TT51-1, Kangyou97, Kefeng6, Kefeng8, KMD1, Bar68-1, and Bar66-1. Only the positive samples contained the target elements, and amplification signals were not observed for the non-GM rice (Table 3).

The GM rice materials used in the application assay are currently undergoing testing and are therefore not yet available for human consumption. Many other GM rice varieties are also in development. However, trace amounts of these GM varieties have been found in the food supplies in Europe and China [33]. Our test results are consistent with databases and other references, and the amplification results indicated that the pBJGMM001 plasmid developed in this study is suitable for use in practical analyses of rice samples.

## 4. Conclusions

Rice is one of the most important food crops worldwide, and most people in developing countries depend on rice as a staple food for nutrition and caloric intake. Due to the importance of rice to humans, major efforts have been made to improve its properties through genetic alterations [11]. The newly developed screening plasmid pBJGMM001 permits greater coverage of food and feed products that may contain GM ingredients. The results of the tests performed in the current study can be compared with the results obtained using existing PCR targets in the Chinese National Standards. The plasmid provides better coverage of the GM elements that could be present in a sample and will facilitate advancements in the detection of unauthorized/unknown GM rice. The reduced need for subsequent identification tests makes the use of this plasmid a cost-beneficial strategy. Indeed, this novel plasmid represents an upgrade due to its many useful screening elements, including promoter and terminator fragments. pBJGMM001 will be certified as a reference material in China. The data reported here regarding GM rice in China and GMOs are valuable tools that can assist in the detection of accidentally introduced unauthorized GM events in the global food and supply chain. The reported plasmid facilitates efficient, rapid, and cost-effective preliminary screening by eliminating the need for the development of specific testing methodologies for GM rice.

pBJGMM001 was designed after considerable investigation and will be used as a certified reference material. The detection coverage of pBJGMM001 reached 96% in all GM rice lines in China. Furthermore, the developed assays were successfully used to test six practical samples from different GM rice lines. All of the results indicated that the established plasmid is a convenient, rapid, and low-cost method for the routine detection of GM rice. The sequence of pBJGMM001 is available from NCBI under accession number KR493382.

## Supplementary Material

Supplementary Table S1: The investigation results of the status of genetically modified rice in China from 1989 to 2014. We show the total list of papers published on development of GM rice varieties is approximately 250.

## Figures and Tables

**Table 1:** Primer information for pBJGMM001.

Target	Primers	Sequences (5′-3′)	Amplicon (bp)	Source
SPS	SPS-F	ATCTGTTTACTCGTCAAGTGTCATCTC	287	MOA 1861-1-2012 [26]
	SPS-R	GCCATGGATTACATATGGCAAGA		

PEPC	PEPC-F	TCCCTCCAGAAGGTCTTTGTGTC	271	MOA 1861-1-2012 [26]
	PEPC-R	GCTGGCAACTGGTTGGTAATG		

T-NOS	NOS-F	GAATCCTGTTGCCGGTCTTG	180	MOA 953-6-2007 [27]
	NOS-R	GAATCCTGTTGCCGGTCTTG		

CaMV35S	35S-F	GCTCCTACAAATGCCATCATTGC	195	MOA 953-6-2007 [27]
	35S-R	GATAGTGGGATTGTGCGTCATCCC		

Ubi	Ubi-F	CCGTAATAAATAGACACCC	314	SN/T 1943-2007 [28]
	Ubi-R	AACACTGGCAAGTTAGCAAT		

Bar	Bar-F	GCTGCCAGAAACCCACGTCAT	430	SN/T 1197-2003 [29]
	Bar-R	ACCATCGTCAACCACTACACCG		

Hpt	Hpt-F	TCGCCTCGCTCCAGTCAATG	472	MOA 1782-2-2012 [30]
	Hpt-R	GTTCACAGGGTGTCACGTTGC		

**Table 2:** Probability statistics of the screening detection of GM rice. P-35S, cauliflower mosaic virus 35S promoter; T-NOS, *Agrobacterium tumefaciens* nopaline synthase terminator; Hpt, hygromycin phosphotransferase gene; Ubi, ubiquitin gene; Bar, bar gene.

Elements	Frequency	Total	Coverage
P-35S	181	250	72.40%
P-35S + T-NOS	209	250	83.60%
P-35S + T-NOS + Hpt	228	250	91.20%
P-35S + T-NOS + Hpt + Ubi	237	250	94.80%
P-35S + T-NOS + Hpt + Ubi + Bar	240	250	96.00%

**Table 3:** Results of the practical screening test. “+” indicates theoretically positive; “−” indicates theoretically negative.

Events	Targets in pBJGMM001						
	SPS	PEPC	P-CaMV35S	T-NOS	Bar	Ubi	Hpt
TT51-1							
Elements	+	+	−	+	−	−	−
Results	+	+	−	+	−	−	−
Kangyou 97							
Elements	+	+	−	+	−	−	+
Results	+	+	−	+	−	−	+
Kefeng6							
Elements	+	+	+	+	−	+	+
Results	+	+	+	+	−	+	+
Kefeng8							
Elements	+	+	−	+	−	+	−
Results	+	+	−	+	−	+	−
KMD1							
Elements	+	+	+	+	−	+	+
Results	+	+	+	+	−	+	+
Bar68-1							
Elements	+	+	+	+	+	−	−
Results	+	+	+	+	+	−	−
Bar66-1							
Elements	+	+	+	+	+	−	−
Results	+	+	+	+	+	−	−
Non-GM							
Elements	+	+	−	−	−	−	−
Results	+	+	−	−	−	−	−
